# Pyrazolones metabolites are relevant for identifying selective anaphylaxis to metamizole

**DOI:** 10.1038/srep23845

**Published:** 2016-03-31

**Authors:** Adriana Ariza, Elena García-Martín, María Salas, María I. Montañez, Cristobalina Mayorga, Natalia Blanca-Lopez, Inmaculada Andreu, James Perkins, Miguel Blanca, José A. G. Agúndez, María J. Torres

**Affiliations:** 1 Research Laboratory, IBIMA-Regional University Hospital of Malaga-UMA, Malaga, Spain; 2 Department of Pharmacology, University of Extremadura, Caceres, Spain; 3 Allergy Unit, IBIMA-Regional University Hospital of Malaga-UMA, Malaga, Spain; 4 BIONAND-Andalusian Centre for Nanomedicine and Biotechnology, Malaga, Spain; 5 Allergy Service, Infanta Leonor Hospital, Madrid, Spain; 6 Unidad Mixta de Investigación IIS La Fe-UPV, Hospital La Fe, Valencia, Spain

## Abstract

Non-steroidal anti-inflammatory drugs (NSAIDs) are the most common cause of hypersensitivity reactions, with pyrazolones the most frequent drugs inducing selective reactions. Immediate selective hypersensitivity to pyrazolones is thought to be mediated by specific-IgE. Sensitivity of *in vitro* diagnostic tests is low and this may be due to the incomplete characterization of the structures involved. Here we investigated whether main metabolites of metamizole (dipyrone) in human could be involved in the immune response using the basophil activation test (BAT). We studied subjects with confirmed selective immediate hypersensitivity to metamizole and performed BAT with metamizole and its metabolites: 4-methylamino-antipyrine (MAA), 4-aminoantipyrine (AA), 4-acetylamino-antipyrine (AAA) and 4-formylamino-antipyrine (FAA). BAT results showed an increase of positive results from 37.5% to 62.5% using metamizole plus metabolites as compared with the BAT carried out only with the parent drug, demonstrating that metamizole metabolites have a role in the reaction and can induce specific basophil activation in patients with immediate hypersensitivity to this drug. Our findings indicate that pyrazolone metabolites are useful for improving the *in vitro* diagnosis of allergic reactions to metamizole.

Adverse drug reactions (ADRs) constitute an important public health issue, causing 3 to 6% of all hospital admissions and occur in 10 to 15% of hospitalized patients^1^. Non-steroidal anti-inflammatory drugs (NSAIDs) are the most common cause of hypersensitivity reactions^2^, including non-immunologically and immunologically mediated reactions, with an important number of reactions induced by a specific immunological mechanism.

The mechanism involved in non-immunological reactions is believed to be based on the inhibition of cyclooxygenase (COX) enzymes in NSAID-sensitive patients^3^ and leads to an exacerbated production of leukotrienes. Patients react to different, non-chemically related NSAIDs^4^. There are no validated *in vitro* diagnostic tests for evaluating these patients.

The mechanism involved in immunological reactions, named single NSAIDs-induced reactions or selective reactions^5^, may be either IgE mediated (immediate reactions) or T-cell dependent (delayed reactions). The most common drugs involved in these reactions are betalactam antibiotics^6^. In NSAIDs allergic reactions, patients react to a single drug or drugs from the same chemical class, having good tolerance to other chemically unrelated NSAIDs^7^,^8^. Focusing on the immediate selective reactions mediated by IgE, drugs commonly involved include acetylsalicylic acid (ASA)^9^, COX-2 selective inhibitors^10^, diclofenac^11^, ketorolac^12^, pyrazolones^13^,^14^,^15^,^16^,^17^, and the analgesic paracetamol^18^, although responses can be caused by all available NSAIDs. Pyrazolones are the most frequent drugs that induce selective reactions, however rather few studies have been carried out to determine if, in addition to parent drugs, their metabolites can also be recognised by IgE antibodies.

The diagnosis of selective allergic reactions to pyrazolones is mainly based on the clinical history, skin testing and/or drug provocation test (DPT)^17^. Skin testing has low sensitivity and the potential risk of eliciting an anaphylactic response^17^. The reason for the low sensitivity may be due to the fact that rather drug metabolites can elicit the anaphylactic reactions. Metamizole ([N-(1,5-dimethyl-3-oxo-2-phenylpyrazolin-4-yl)-N-methylamino] methanesulfonate, drug bank id. no. DB04817) is hydrolysed in the intestine to 4-methylaminoantipyrine (MAA) after administration, and it is rapidly and almost completely absorbed from the gastrointestinal tract. MAA is further metabolized in the liver by demethylation to 4-aminoantipyrine (AA) and by oxidation to 4-formylaminoantipyrine (FAA). AA is further acetylated by the polymorphic N-acetyltransferase-2 system to 4-acetylaminoantipyrine (AAA)^19^. Because metamizole is rapidly metabolized after administration, it is reasonable to hypothesize that part of the allergic reactions could be due to drug metabolites rather than the drug itself (Fig. 1). Specific basophil activation in subjects with immediate hypersensitivity to metamizole has been used as a proof to infer that these patients may have specific IgE antibodies^14^,^20^. In subjects with immediate positive skin test, parallel positive basophil activation can be observed as occurs with IgE responses to other drugs^21^,^22^,^23^. Basophil activation test (BAT) constitutes a valuable *in vitro* method which safely substitutes to the direct application of the drug and additionally provides “proof of mechanism” in these reactions^24^. The procedure is based on the determination of basophil activation in presence of specific haptens by measuring CD63 expression on the cell surface. This procedure has been used for the diagnosis of IgE responses to betalactams^22^ and muscle relaxants^21^ as well as other drugs^23^.

The aim of this study was to analyze whether metamizole metabolites could be recognised by IgE bound to basophil surface and induce their activation in subjects with confirmed immediate hypersensitivity to these drugs.

## Experimental Design

In order to perform this pilot study, a group of patients with a confirmed selective immediate allergic reaction to metamizole, and a group of tolerant controls were selected. The diagnosis of allergy was based on an immediate skin test, since it is the only *in vivo* method that confirms an IgE mechanism when positive.

With the aim of evaluating which structures derived from metamizole were recognized by IgE from allergic patients to this drug, BAT was performed using metamizole and four metabolites purified from human urine samples (MAA, AA, AAA and FAA) at different concentrations. The optimal cut-off point that corresponds to the best sensitivity and specificity for the native metamizole and its metabolites was calculated by receiver operating characteristic (ROC) curve analysis. With this method we needed to make a balanced decision between sensitivity and specificity. In the case of drug allergy, and especially in severe reactions, the decision must be chosen to increase the sensitivity in spite of a loss of specificity. Finally, in order to confirm an IgE-mediated basophil activation, the phosphatidylinositol 3-kinase (PI3-K) inhibitor, wortmannin (WTM), was used in BAT for those metabolites that showed to induce relevant specific basophil activation.

## Results

### Patients

The study included 16 patients, 13 women and 3 men, the median age was 57.5 years (interquartile range (IR): 46.5–63.5 years), 8 had developed anaphylaxis and 8 urticaria accompanied or not by angioedema, and the median interval between the reaction and the study was 109 days (IR: 59.25–365 days). All patients were skin test positive in immediate reading to metamizole (7 by prick and 9 by intradermal test) and demonstrated good tolerance to ASA, therefore they were classified as selective reactors to metamizole (Table 1). The study also included a control group formed by 15 non-atopic subjects with skin test negative and good tolerance to metamizole.

### Basophil activation test

Dose-response curves studies (0.125 mg/mL–2.5 mg/mL) for the metabolites analyzed showed that the lowest concentration tested (0.125 mg/mL) was not relevant for all the metabolites and the highest concentration (2.5 mg/mL) produced non-specific basophil activation only for the metabolite MAA (Fig. 2). BAT results obtained for the optimal concentrations of metamizole and their metabolites are shown in Table 2 expressed as stimulation index (SI). We found positive results to metamizole in 6 patients, to metamizole metabolites in 10 patients, 8 to MAA, 3 to AA, 2 to AAA and 1 to FAA. A representative positive case (P4) analyzed by flow cytometry is shown in Fig. 3. Considering the parent drug and their metabolites we found 37.5% of positive cases to metamizole, 50% to MAA, 18.75% to AA, 12.5% to AAA, 6.25% to FAA. The percentage of positive cases increased to 62.5% when metamizole and/or any of the four metabolites were considered. Two of the healthy controls showed positive results, one of them to MAA (1.25 mg/mL) and the other one to AAA (1.25 mg/mL), with a specificity of the test of 86.7%.

We tried to confirm the involvement of specific IgE by using a PI3-K inhibitor, WTM. BAT inhibition with WTM was performed with samples from four positive patients to MAA and found a mean percentage of inhibition of basophil activation of 73.96% for MAA and of 85.16%, for the positive control anti-IgE. As expected, no significant inhibition was observed with the positive control N-formyl-Met-Leu-Phe (fMLP), which is a positive control of non-mediated IgE basophil activation (Fig. 4).

## Discussion

NSAIDs are the drugs most frequently involved in hypersensitivity drug reactions^2^. These reactions can be mediated by specific immunological mechanisms (allergic reactions) and non-specific immunological mechanisms (non-allergic hypersensitivity)^25^. Their diagnosis is usually complex, requiring trained personnel and appropriate resources^26^. Focusing on selective allergic reactions, pyrazolones are the NSAIDs most frequently involved in IgE-mediated reactions^14^,^17^, and their diagnosis is mainly based on clinical history and the confirmation of ASA tolerance by DPT, which consumes both time and resources and runs the risk of potentially harming the patient^17^. Skin tests and *in vitro* tests such as BAT are also used for diagnosis with variable results in different studies^14^,^20^,^27^,^28^. Gamboa *et al*.^14^ reported the value of BAT in a group of subjects with immediate selective allergic reactions to metamizole, where sensitivity was 42.3% and specificity was 100%. Similar results were observed in another study performed by Gomez *et al*.^20^ in which the sensitivity of BAT was 54.9% and specificity was 85.7%. The slight difference in sensitivity may be due to the mean time interval between the reaction and study, which was longer in the study of Gamboa (17 months) than of Gomez (13 months) and the fact that the percentage of patients with anaphylaxis was higher in the latter (57.7% *vs.* 74.5%).

These tests used the parent drug only, limiting their power to detect the relevant specific IgE antibodies. In the present work we verified if, in addition to metamizole, metabolites of this drug could induce basophil activation. Experimental evidence indicates that several pyrazolone structures can be recognised by a given antibody, indicating cross-reactivity^13^. Focusing on metamizole, different metabolites formed either by alkaline hydrolysis^29^ or by biotransformation^30^,^31^,^32^ have been identified, although IgE recognition of these structures has not previously been demonstrated. In this study we have found that the metabolite MAA was recognised by 50% of the positive cases tested, and in fact elicited more positive cases than native metamizole (37.5%) (with all positive cases to metamizole also being positive to MAA). Moreover, MAA was the metabolite that induced higher SI with positive results at both concentrations used in the assay (0.25 and 1.25 mg/mL). However, low or non-positive responses were detected for the other metabolites (AA, AAA and FAA) analyzed.

Considering the response to the native molecule, metamizole, the sensitivity achieved here was lower than in previous studies^14^,^20^, this may be due to the clinical characteristics of the patients studied: the number of patients with an anaphylactic reaction was lower in the present study (50% of patients with a history of anaphylaxis reaction compared with 58%^14^ and 75%^20^ respectively for the previous studies).

It is thought that the molecules that induce basophil activation require covalent binding to proteins. In fact it has been suggested that pyrazolones become antigenic after binding to biomacromolecules following metabolic activation^33^. Metamizole is rapidly hydrolysed to MAA and is almost completely absorbed in this form^34^, consequently this metabolite is more exposed to protein binding than metamizole, and as a result is more likely to induce an immune response. This could explain why greater IgE recognition is found using a metabolite than with the parent drug. Furthermore, MAA is the primary *in vivo* metabolite of metamizole. Although none of the metabolites are bound to more than 60% of plasma proteins^34^, the binding of MAA and AA is relatively higher than that of FAA and AAA, as expected from their chemical structure^35^. This is in agreement with the basophil activation results in this study, where activation was mainly obtained with MAA and AA.

There is some controversy as to whether selective reactions to NSAIDs are IgE-mediated because specific IgE antibodies had not been identified directly. However, in the case of immediate reactions to metamizole, the results of skin testing and BAT indirectly support that these reactions are IgE-mediated. Further support is given by the tendency for BAT results to decrease over time in these patients^36^. In order to confirm the specific IgE mediation, we have followed the previous approach of BAT inhibition with WTM^37^. WTM is a potent and specific inhibitor of PI3-K and has been used to demonstrate the role of this enzyme in diverse signal transduction processes, such as the regulation of histamine release by basophils and mast cells^38^. We observed that WTM induced BAT inhibition when basophils were stimulated with the positive control anti-IgE and with the metabolite MAA, but not with the positive control fMLP, which induces basophil activation through a non IgE-mediated mechanism.

Given these findings, the next step would be to put these metabolites with the appropriate carriers in different solid phases and test for the presence of specific IgE antibodies using immunoassays. To our knowledge, specific IgE antibodies have only been detected *in vitro* for propyphenazone^15^, furthermore these results have not been validated by other groups, and have not been demonstrated for other pyrazolones^8^. Further studies that aim to design the appropriate conjugation of the drug metabolite to the candidate carrier molecule are needed. Whether these compounds have preferential protein conjugation as occurs with amoxicillin^39^ must also be studied in order to select the appropriate drug carrier or drug adduct for detection of specific IgE antibodies.

In summary, we have shown that metamizole metabolites can be specifically recognised by IgE antibodies bound to the surface of basophils, inducing their activation in subjects with anaphylactic reactions to metamizole. These results deserve further study with an increased number of patients in order to further explore whether MAA is a relevant structure recognized by IgE antibodies. Moreover, more studies are needed to identify the full range of metamizole metabolites and to assess their utility in testing for metamizole allergy. This is the first study demonstrating the role of metamizole metabolites in immediate reactions and the findings obtained here support the hypothesis that drug metabolites may underlie allergic reactions. This may be a major cause of the lack of sensitivity that has been observed when BAT is performed using the parent drug only. It is highly recommended to design BAT to mimic as much as possible the *in vivo* events that occur after drug administration, including drug biotransformation, in order to improve the sensitivity of the test and to make it clinically viable.

## Methods

### Patients

We selected patients with confirmed immediate allergic reactions to pyrazolones. The diagnosis was established by the presence of clinical symptoms of anaphylaxis or immediate urticaria after a pyrazolone derivative intake (in all cases metamizole) and a positive skin test (Table 1). To rule out an idiosyncratic reaction with NSAIDs cross-intolerance, we assessed the response to ASA in all cases. As controls, we included subjects with good tolerance to both metamizole and ASA, confirmed by a negative DPT.

The study was conducted according to the Declaration of Helsinki principles and was approved by the Malaga Research Ethics Committee. All subjects were informed orally about the study and signed an informed consent form.

### Skin testing

Skin testing was carried out as previously described^14^,^20^, using metamizole 0.1 mg/mL (Boehringer Ingelheim, Barcelona, Spain). In the skin prick tests, a wheal larger than 3 mm with a negative response to the control saline was considered positive. In the intradermal tests, the wheal area was marked initially and 20 min after testing, and an increase in diameter greater than 3 mm was considered positive^40^.

### Drug provocation test

To exclude a cross-intolerant response we carried out a single-blind procedure in all patients, as previously described^25^, with some modifications. Increasing doses of ASA (50, 100, 150 and 200 mg) (Bayer-Hispania SL, Barcelona, Spain) were administered orally at 90 min intervals. If cutaneous, respiratory or systemic symptoms appeared the procedure was stopped, the symptoms evaluated and appropriate treatment administered. If no symptoms occurred during the DPT, the therapeutic dose was reached. This was followed 24 h later by a dose of 500 mg of ASA every 12 h for 2 days.

### Metabolites obtention

**Figure 1:**
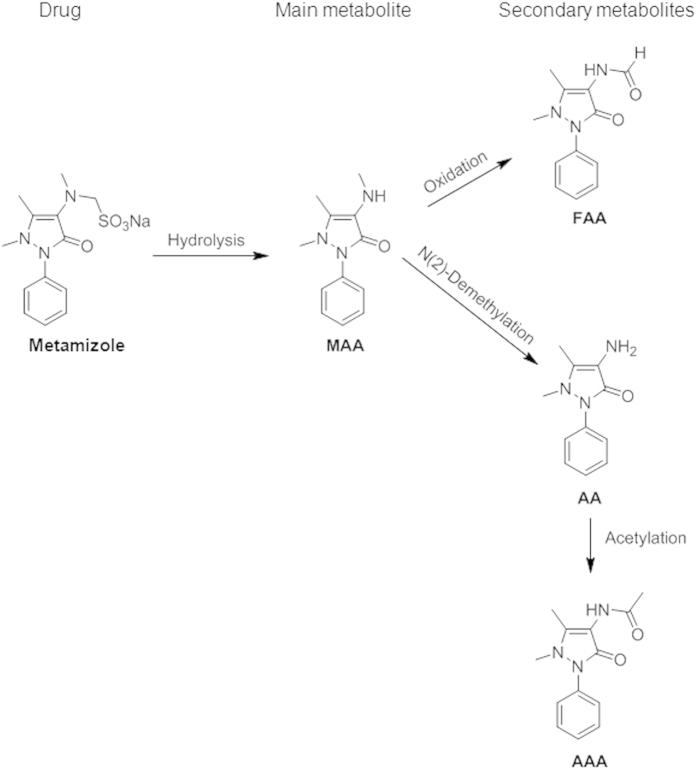
Main metabolic pathways of metamizole in humans. Metabolites are: 4-methylamino antipyrine (MAA), 4-aminoantipyrine (AA), 4-acetylamino antipyrine (AAA) and 4-formylamino antipyrine (FAA).

Metamizole metabolites were purified from human urine samples, obtained from healthy individuals who received an oral dose of 575 mg of metamizole (Nolotil®, Boehringer Ingelheim, Barcelona, Spain), with the exception of 4-aminoantipyrine (AA) which was purchased from SIGMA chemical Co. (St. Louis, MO, USA). Because the vast majority of metabolites are recovered in urine in the first 24 h^30^, urine was collected from subjects 24 h after the administration of the drug, placed in sterile plastic containers and frozen at −80 °C until use. Thawed urine samples were filtered through a 0.8 μm Nalgene filter (Nalgene Co., Rochester, NY, USA), alkalinized and chloroform-extracted as described elsewhere^30^,^41^. After evaporation under a nitrogen stream, residues were dissolved in the mobile phase and injected onto a Chromolit performance RP-18e (100 × 4.6 mm) HPLC column (Merck AG, Darmstad, Germany). The equipment consisted of a Hitachi LaChrom Elite HPLC, equipped with a L-2130 solvent delivery module, a refrigerated autosampler L-2200 and an ultraviolet-visible detector L-2420 (Hitachi, Krefeld, Germany). The mobile phase was water:methanol:triethylamine:acetic acid (70.9:27.7:0.9:0.5) and the flow rate was 1 mL/min. Column effluents were monitored at 254 nm and effluent fractions were collected in parallel with the absorption peaks corresponding to the metabolites, which eluted in the following order: FAA, AAA, AA and MAA (Fig. 1). Pooled fractions for each metabolite were extracted with chloroform by shaking vigorously for 5 min and centrifuged for 5 min at 2,000 × *g*. The organic phase was transferred to fresh tubes and dried by evaporation under a nitrogen stream. Then, the tubes containing the evaporated metabolites were closed to maintain the nitrogen environment, and kept frozen at −80 °C until use. The concentration and purity of the metabolites was checked by HLPC analysis as described elsewhere^30^,^31^ by comparing the retention times and UV-VIS spectrum with pure MAA, FAA and AAA metabolites kindly provided by Drs. Bremer and Eckert (Hoechst Aktiengesellschaft; Radiochemistry laboratory, Frankfurt am Main, Germany)

### Basophil activation test

**Figure 2:**
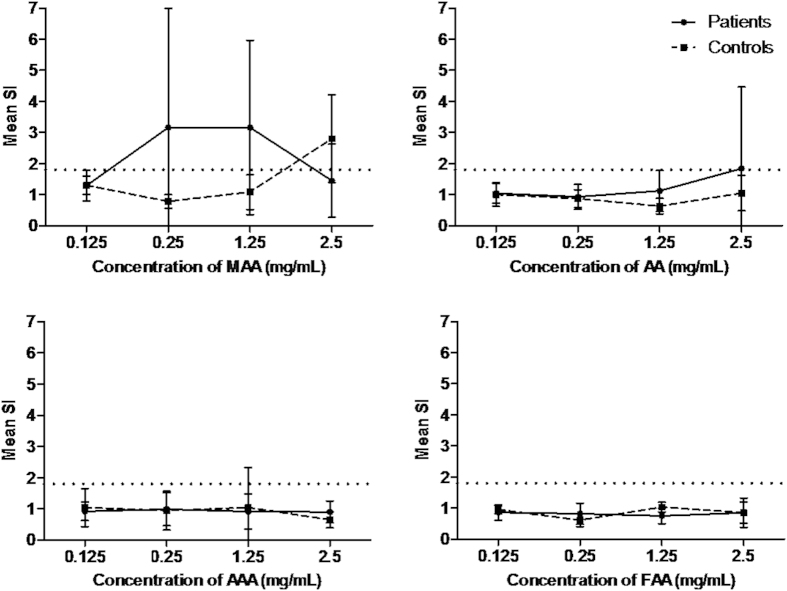
Dose-response curve for basophil activation test performed with metamizole metabolites. 4-methylamino antipyrine (MAA), 4-aminoantipyrine (AA), 4-acetylamino antipyrine (AAA) and 4-formylamino antipyrine (FAA). Results are expressed as the mean + SD of stimulation index (SI). Patients (N = 16) and healthy controls (N = 15).

**Figure 3:**
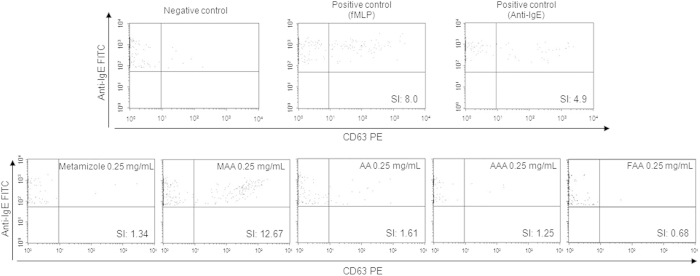
Representative dot plot graph of patient 4 showing basophil activation with negative control, positive controls (fMLP and anti-IgE), metamizole and metamizole metabolites: 4-methylamino antipyrine (MAA), 4-aminoantipyrine (AA), 4-acetylamino antipyrine (AAA) and 4-formylamino antipyrine (FAA) at 0.25 mg/mL. The figure also shows the stimulation index (SI) values for each determination.

**Figure 4:**
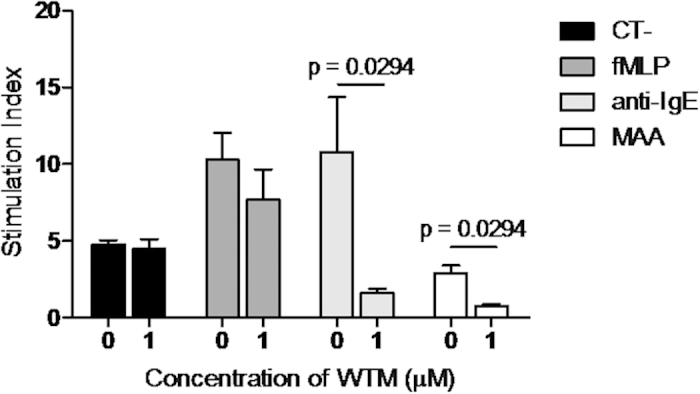
Results of basophil activation inhibition with wortmannin (WTM) after stimulation with 4-methylamino antipyrine (MAA) or positive controls (anti-IgE and fMLP). Bars show the mean value of stimulation index obtained for non-inhibited and WTM inhibited basophil activation. Significant differences (p < 0.05) are indicated in the graph.

This assay was performed as previously described with some modifications^20^,^22^. Whole blood, extracted in collection tubes with acid citrate dextrose, was incubated with stimulation buffer (0.78% NaCl; 0.037% KCl (w/v); 0.078% CaCl_2_ (w/v); 0.033% MgCl_2_ (w/v); 0.1% HSA (w/v); 1 M HEPES and 10 μL/mL IL-3) for 10 min at 37 °C. Then, metamizole (Sigma-Aldrich, St. Louis, MO, USA) and their metabolites (MAA, AA, AAA, FAA) obtained as described above, were added at different concentrations chosen from dose-response curves and cytotoxicity studies (Fig. 2)^14^,^20^, and were incubated for 30 min at 37 °C. Phosphate buffer saline was used as a negative control, and two positive controls were used: chemotactic peptide fMLP (2 μM) (Orpegen Pharma, Heidelberg, Germany) and anti-human IgE (0.05 mg/mL) (Dako, Glostrup, Denmark). After that, samples were incubated on ice for 5 min to stop the degranulation process and samples were incubated with monoclonal antibodies anti-CD63-phycoerythrin and anti-IgE–fluorescein isothiocyanate (Caltag Laboratories, Burlingame, CA, USA) for 15–20 min at 4 °C. Finally, lysing solution (BD Biosciences, San Jose, CA, USA) was used to lyse red cells. Cells were analyzed in a FACSCalibur flow cytometer (BD Biosciences, San Jose, CA, USA), acquiring at least 500 basophils per sample. Results were considered as positive when the SI, calculated as the ratio between the percentage of CD63 on cell surface of basophils stimulated with the haptens and the spontaneous basophil activation, was >1.8 for at least one of the hapten concentrations used^22^.

In order to confirm that the basophil activation was IgE-mediated we analysed the WTM inhibitory effect, since WTM is a specific inhibitor of PI3-K which is involved in the IgE basophil activation^38^,^42^. This inhibition assay was performed by incubating the samples at 37 °C with WTM (Sigma-Aldrich, St. Louis, MO, USA) at 1 μM (previously selected as the optimal concentration)^37^ for 5 min after the incubation with the stimulation buffer and following the protocol as described above.

### Statistical analysis

Comparisons for quantitative variables without a normal distribution were carried out by the Mann–Whitney test. ROC curve analyses were performed to calculate the optimal cut-off value corresponding to the best sensitivity and specificity. P values < 0.05 were considered statistically significant.

## Additional Information

**How to cite this article**: Ariza, A. *et al*. Pyrazolones metabolites are relevant for identifying selective anaphylaxis to metamizole. *Sci. Rep.* **6**, 23845; doi: 10.1038/srep23845 (2016).

## Figures and Tables

**Table 1:** Clinical characteristics and results of the allergological work up.

Pat	Age	Sex	Interval reac (min)	Interval study (days)	Culprit drug	Clinical entity	Skin test (mm)	ASA DPT
P1	57	F	30	61	Metamizole	Urticaria	SPC (4 × 5)	(–)
P2	34	F	45	35	Metamizole	Anaphylaxis	SPC (3 × 4)	(–)
P3	67	F	15	98	Metamizole	Anaphylaxis	ID (3 × 5)	(–)
P4	24	M	15	120	Metamizole	Urticaria	ID (3 × 4)	(–)
P5	51	F	45	47	Metamizole	Urticaria	SPC (5 × 6)	(–)
P6	42	F	30	30	Metamizole	Urticaria	ID (4 × 4)	(–)
P7	61	F	30	92	Metamizole	Urticaria	SPC (4 × 3)	(–)
P8	58	M	15	54	Metamizole	Anaphylaxis	ID (4 × 5)	(–)
P9	72	F	30	365	Metamizole	Urticaria+angioedema	ID (3 × 4)	(–)
P10	52	F	30	365	Metamizole	Urticaria	ID (3 × 5)	(–)
P11	65	M	30	365	Metamizole	Urticaria	ID (5 × 6)	(–)
P12	71	F	15	90	Metamizole	Anaphylaxis	SPC (5 × 4)	(–)
P13	40	F	40	180	Metamizole	Anaphylaxis	ID (4 × 3)	(–)
P14	63	F	20	365	Metamizole	Anaphylaxis	SPC (3 × 4)	(–)
P15	61	F	35	365	Metamizole	Anaphylaxis	ID (4 × 5)	(–)
P16	48	F	10	365	Metamizole	Anaphylaxis	SPC (3 × 4)	(–)

Pat: patient; Reac: reaction; ASA: acetylsalycilic-acid; DPT: drug provocation test; F: female; M: male; SPC: skin prick test; ID: intradermal test.

**Table 2:**
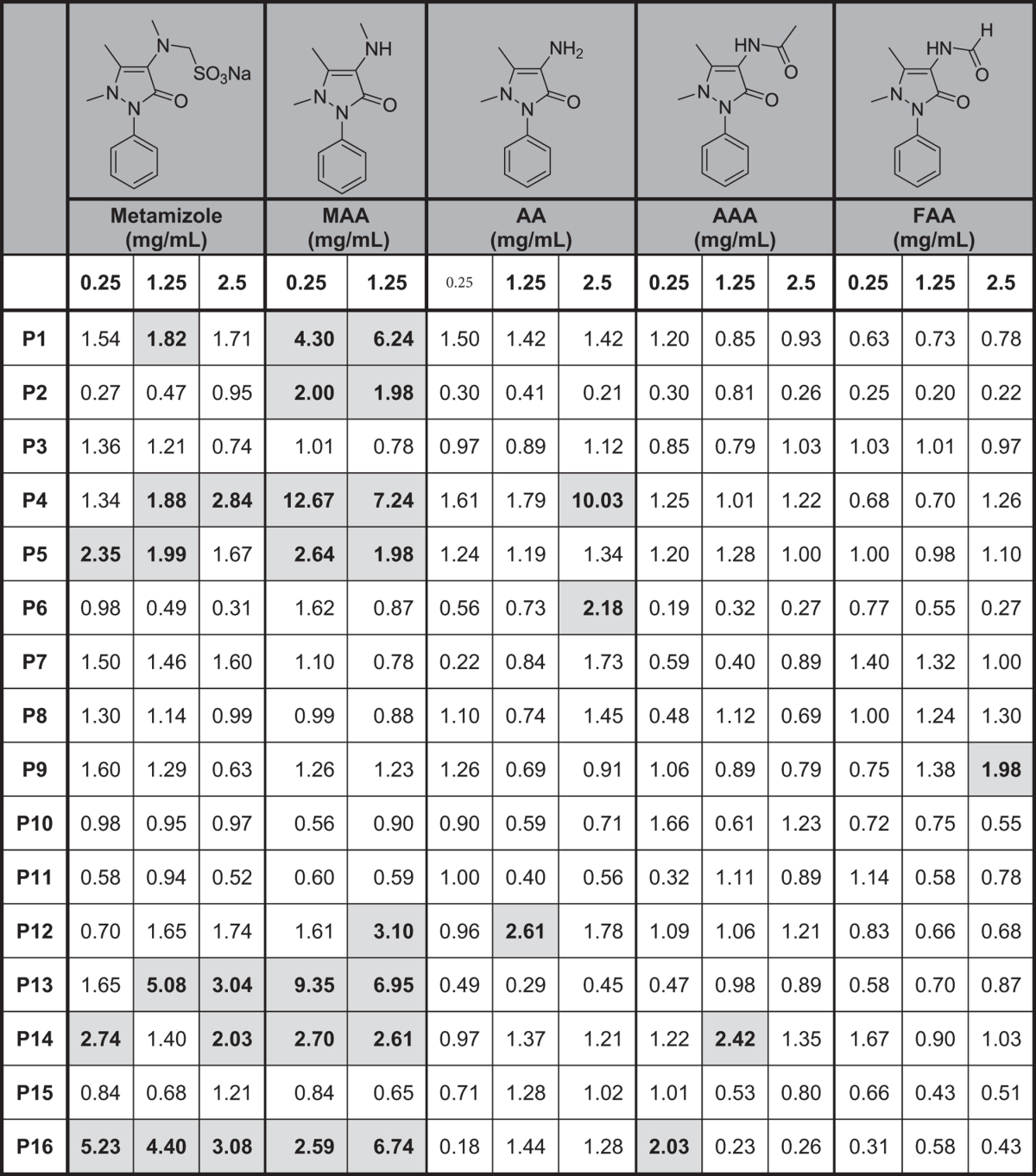
Results of basophil activation to metamizole and metamizole metabolites at different concentrations (0.25-2.5 mg/mL) expressed as stimulation index (SI) values.

Cut-off for considering positivity >1.80. MAA: 4-methylamino antipyrine; AA: 4-aminoantipyrine; AAA: 4-acetylamino antipyrine; FAA: 4-formylamino antipyrine.
